# Ferulic Acid Suppresses Amyloid *β* Production in the Human Lens Epithelial Cell Stimulated with Hydrogen Peroxide

**DOI:** 10.1155/2017/5343010

**Published:** 2017-03-20

**Authors:** Noriaki Nagai, Sachiyo Kotani, Yu Mano, Akina Ueno, Yoshimasa Ito, Toshio Kitaba, Takumi Takata, Noriko Fujii

**Affiliations:** ^1^Faculty of Pharmacy, Kindai University, 3-4-1 Kowakae, Higashi-Osaka, Osaka 577-8502, Japan; ^2^Kitaba Society, Social Welfare Corporation, 2-2-15 Koyodai, Tondabayashi, Osaka 584-0082, Japan; ^3^Research Reactor Institute, Kyoto University, Asashironishi Kumatori, Sennan, Osaka 590-0494, Japan

## Abstract

It is well known that oxidative stresses induce the production of amyloid *β* (A*β*) in the brain, lens, and retina, leading to age-related diseases. In the present study, we investigated the effects of ferulic acid on the A*β* levels in H_2_O_2_-stimulated human lens epithelial (HLE) SRA 01/04 cells. Three types of A*β* peptides (A*β*_1-40_, A*β*_1-42_, and A*β*_1-43_) were measured by ELISA, and the levels of mRNA for the expressed proteins related to A*β* production (APP, BACE1, and PS proteins) and degradation (ADAM10, NEP, and ECE1 proteins) were determined by quantitative real-time RT-PCR. H_2_O_2_ stimulation augmented gene expression of the proteins related to A*β* production, resulting in the production of three types of A*β* peptides. Treatment with 0.1 *μ*M ferulic acid attenuated the augmentations of gene expression and production of the proteins related to the secretion of three types of A*β* peptides in the H_2_O_2_-stimulated HLE cells. These results provided evidence of antioxidative functions of ferulic acid for lens epithelial cells.

## 1. Introduction

A sequential proteolytic processing of amyloid precursor protein (APP) cleaved by *β*-secretase (*β* site APP cleaving enzyme, BACE1) [1] and *γ*-secretase [a presenilin complex (PS)] [2] leads to the production of amyloid *β* (A*β*) peptides. The cleavage of APP at different positions by *γ*-secretase mainly produces three A*β* peptides, A*β*_1–40_, A*β*_1–42_, and A*β*_1–43_ peptides [3–7]. The most abundant variant of A*β* is A*β*_1–40_, but A*β*_1–42_ and A*β*_1–43_, the longer forms, are more neurotoxic than A*β*_1–40_. In addition, the neurotoxicity of A*β*_1–43_ is higher than that of A*β*_1–42_, although the accumulation of A*β*_1–43_ is lower than that of A*β*_1–42_ [7]. However, the APP cleaved by *α*-secretase (a disintegrin and metalloprotease domain protease 10, ADAM10) generates nonamyloidogenic soluble APP*α* [8–10]. Neprilysin (NEP) [11] and endothelin converting enzyme (ECE) [12] degrade the A*β* peptide. The production of A*β* is a normal process; however, the overproduction of A*β*, or an increased proportion of A*β*_1–42_ and A*β*_1–43_, appears to cause an early onset of cataract and Alzheimer's disease [13, 14].

Cataract represents a disease of increasing lens opacity, and it has been reported that A*β* accumulation in the human lens causes lens opacification [15–18]. In addition, there have been several reports that oxidative stress can increase the accumulation and toxicity of A*β* peptides in the lens, retina, and brain and that enhanced A*β* accumulation leads to oxidative stress [14, 19–24]. Therefore, it is expected that drugs that inhibit the effect of A*β* peptides and lower oxidative stress can be used as anticataract eye drops.

Ferulic acid (4-hydroxy-3-methoxycinnamic acid) is a widely distributed constituent of plants and is used as an antioxidant to prevent the oxidation of substrates such as lipids, proteins, DNA, and carbohydrates [25, 26]. Ferulic acid has been shown to possess some scavenging activity toward hydroxyl radicals and peroxynitrite [27, 28]. Recently, several studies have shown that ferulic acid has beneficial effects against Alzheimer's disease [29–31], diabetes [32], and cancer [33]. Alzheimer's disease is a neurodegenerative disorder, and it had been shown that one of the main pathophysiological features of Alzheimer's disease was the presence of extracellular senile plaques consisting essentially of A*β*, a peptide thought to be a leading cause of neurotoxicity [34]. It was reported that ferulic acid could be a suitable molecule to specifically bind to A*β* and inhibit fibril formation [35]. Furthermore, its compact structure could also be used for specific interactions with A*β* mature fibrils, thereby possibly promoting their destabilization [35]. Ferulic acid inhibits A*β* aggregation, destabilizes preformed A*β* fibrils in vitro [36], and protects cultured neuronal cells against A*β*-induced cytotoxicity [29]. Moreover, long-term administration of ferulic acid protects mice against A*β*-induced learning and memory deficits in vivo [30, 31]. Therefore, ferulic acid may be a useful molecule for the inhibition of A*β* production and accumulation.

In this study, we investigated the changes in A*β* production and accumulation in human lens epithelial (HLE) SRA 01/04 cells stimulated with hydrogen peroxide (H_2_O_2_). In addition, we demonstrated the preventive effects of ferulic acid on A*β* production and accumulation in HLE cells.

## 2. Materials and Methods

### 2.1. Cell Culture and Treatment

The HLE cell line SRA 01/04 was incubated in Dulbecco's Modified Eagle's Medium (DMEM) containing 10 *μ*g/L gentamicin and 10% (v/v) fetal bovine serum (FBS) under humidified air containing 5% CO_2_, at 37°C. Each treatment was usually carried out on the third day after seeding of 0.4 × 10^4^ cells/cm^2^ (at approximately 80% confluency), and the culture medium was changed to non-FBS medium 1 hour before each experiment. The experiment was initiated by changing the medium to fresh medium containing 0–100 *μ*M of H_2_O_2_ and the HLE cells were cultured for 24 hours to produce A*β*_1–40_, A*β*_1–42_, and A*β*_1–43_ (H_2_O_2_-stimulated HLE cells). Ten *μ*M of H_2_O_2_ and 0–30 *μ*M of ferulic acid included in the medium were used for 24-hour cultures to observe the effect of ferulic acid on A*β* production (H_2_O_2_-stimulated HLE cells in the presence of ferulic acid).

### 2.2. Quantitative Real-Time RT-PCR

The experiment was performed as described previously [14, 37]. Briefly, extraction and purification of the total RNA from HLE cells were performed using an RNase-Free DNase kit and an RNeasy Mini kit (Qiagen, Tokyo, Japan), and an RNA PCR kit (AMV Ver. 3.0, Takara Bio Inc., Shiga, Japan) was used for the reaction according to the manufacturer's instructions. Total RNA (1 *μ*g, OD_260_/OD_280_ values > 1.8) was used for the reverse transcription (RT) reaction, and the reaction was performed at 42°C for 15 min, followed by 5 min at 95°C. The PCR reactions were performed using LightCycler® FastStart DNA Master SYBR Green I according to the manufacturer's instructions (Roche Diagnostics Applied Science, Mannheim, Germany), and the quantities of the PCR products were measured fluorometrically in a real-time manner using a LightCycler® DX 400 (Roche Diagnostics Applied Science). In this study, the specific primers (10 pmol) for APP, BACE1, PS1, PS2, ADAM10, NEP, ECE1, or glyceraldehyde-3-phosphate dehydrogenase (GAPDH) were as follows: 5′-TGGTGGGCGGTGTTGTCATA-3′ (forward; FOR) and 5′-TGGATTTTCGTAGCCGTTCTGC-3′ (reverse; REV) for APP; 5′-GCAAGGAGTACAACTATGAC-3′ (FOR) and 5′-AGCTTCAAACACTTTCTTGG-3′ (REV) for BACE1; 5′-ATCATCTGCATAGTCCTCTC-3′ (FOR) and 5′-AGACAGCTTTGATGTTCAAG-3′ (REV) for PS1; 5′-TACAAGTACCGCTGCTACAAGTTC-3′ (FOR) and 5′-GCACTTCCCCAAGGTAGATATAGG-3′ (REV) for PS2; 5′-CACATGATTCTGGAACAGAG-3′ (FOR) and 5′-GTTGTTAAGTTTGTCCCCAG-3′ (REV) for ADAM10; 5′-CTGATATCAACACTCCAAAGC-3′ (FOR) and 5′-TCATCGTAGGTTGCATAGAG-3′ (REV) for NEP; 5′-AGAATGAGATTGTGTTTCCG-3′ (FOR) and 5′-CTATGCCACCAAAGTTTAAGG-3′ (REV) for ECE1; and 5′-TGCACCACCAACTGCTTAGC-3′ (FOR) and 5′-GGCATGGACTGTGGTCATGAG-3′ (REV) for GAPDH. The conditions for PCR were 95°C for 10 min (hot start), 60 cycles of 95°C for 10 s (denaturing), 63°C for 10 s (annealing), and 72°C for 5 s (extension). The differences in the threshold cycles for target groups (APP, BACE1, PS1, PS2 ADAM10, NEP, and ECE1) and GAPDH were used to calculate the levels of mRNA expression in HLE cells.

### 2.3. Measurement of A*β*

The measurements of A*β*_1–40_, A*β*_1–42_, and A*β*_1–43_ levels were performed as previously described [14, 38]. Approximately 2.5 × 10^6^ HLE cells were collected with a cell scraper (Asahi Glass, Tokyo, Japan), homogenized in 250 *μ*L of 70% formic acid, and then centrifuged at 9,100 ×g for 15 min at 4°C. The supernatants were added to 4.75 mL of 1 M Tris base, and the mixtures were used for the measurements of the A*β*_1–40_, A*β*_1–42_, and A*β*_1–43_ peptides. The A*β*_1–40_, A*β*_1–42_, and A*β*_1–43_ levels were measured using the human *β* amyloid (40) ELISA kit (dynamic range, 1–100 pM, Wako, Osaka, Japan), the human *β* amyloid (42) ELISA kit (dynamic range, 0.1–20 pM, Wako), and the human amyloid *β* (1–43) (FL) ELISA kit (dynamic range, 0.51–32.5 pM, IBL, Gunma, Japan) according to the manufacturer's instructions, respectively. The A*β* levels were expressed as pmol/g of protein according to our previous reports. The protein levels in the samples used to determine A*β*_1–40_, A*β*_1–42_, and A*β*_1–43_ were assessed using a Bio-Rad Protein Assay kit (Bio-Rad, Hercules, CA, USA).

### 2.4. Measurement of Cell Viability

HLE cells with or without added H_2_O_2_ and ferulic acid treatments were incubated in 96-well microplates (Iwaki, Chiba, Japan). The viability of the HLE cells was calculated by the Cell Count Reagent SF kit (Nacalai Tesque, Kyoto, Japan) according to the manufacturer's instructions. The absorbance at 450 nm was measured, and the cell viability (%) was represented as the percentage of the absorbance measured for unstimulated HLE cells for each point (normal cells).

### 2.5. Statistical Analyses

All values were presented as mean ± standard error (SE), and statistical differences were evaluated by one-way analysis of variance followed by Dunnett's multiple comparisons.* P* values of less than 0.05 were considered significant.

## 3. Results

### 3.1. The Effect of H_2_O_2_ Stimulation on A*β* Production and Accumulation in HLE Cells

**Figure 1:**
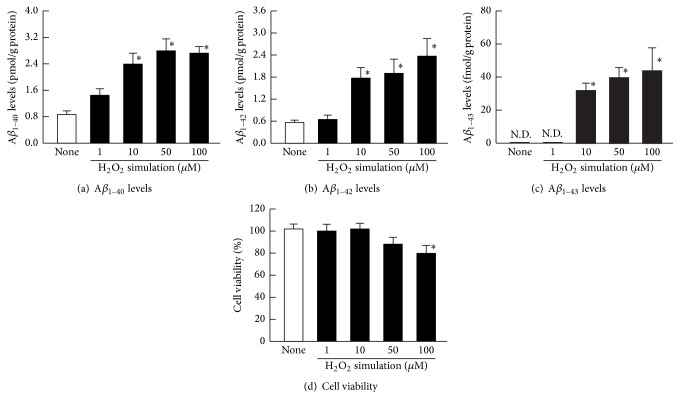
Effect of H_2_O_2_ stimulation on A*β*_1–40_ (a), A*β*_1–42_ (b), A*β*_1–43_ (c), and cell viability (d) in HLE cells. The HLE cells were stimulated by 1–100 *μ*M H_2_O_2_ for 24 h. Open columns (none) = unstimulated HLE cells. Closed columns = H_2_O_2_-stimulated HLE cells. N.D. = not detectable. The data are presented as the means ± SE of 5–8 experiments. ^*∗*^*P* < 0.05, versus none for each category.

**Figure 2:**
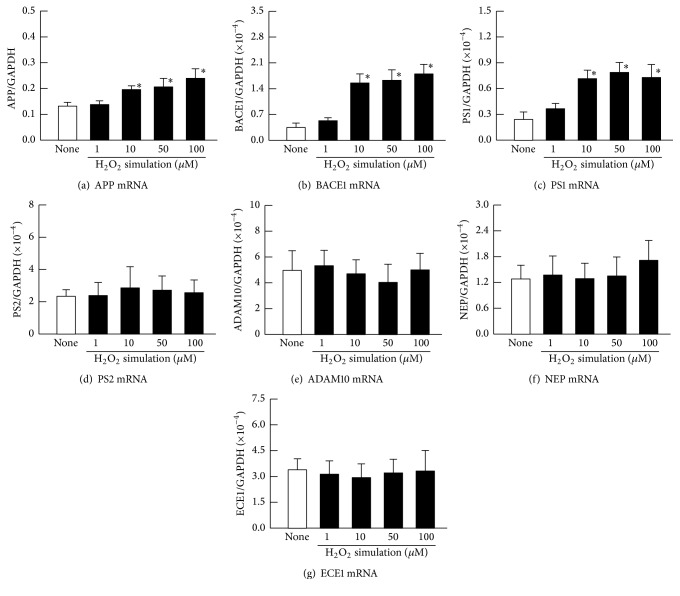
Effect of H_2_O_2_ stimulation on mRNA levels of APP (a), BACE1 (b), PS1 (c), PS2 (d), ADAM10 (e), NEP (f), and ECE1 (g) in HLE cells. The HLE cells were stimulated with 1–100 *μ*M H_2_O_2_ for 24 h. Open columns (none) = unstimulated HLE cells. Closed columns = H_2_O_2_-stimulated HLE cells. The data are presented as the means ± SE of 5–8 experiments. ^*∗*^*P* < 0.05, versus none for each category.

Figures 1(a)–1(c) shows the levels of A*β* accumulation in the H_2_O_2_-stimulated HLE cells. The A*β*_1–40_ and A*β*_1–42_ levels were enhanced with increasing H_2_O_2_ concentration. Although the A*β*_1–43_ was not detected in the unstimulated HLE cells, an accumulation of A*β*_1–43_ was observed with stimulation by 10–100 *μ*M H_2_O_2_.  Figure 1(d) shows the cell viability in the H_2_O_2_-stimulated HLE cells. The cell viability in the 1 *μ*M and 10 *μ*M H_2_O_2_-stimulated HLE cells was similar to that of the unstimulated HLE cells (None). However, the HLE cell viabilities in the presence of 50 *μ*M and 100 *μ*M H_2_O_2_ were decreased, and the cell viability at 100 *μ*M H_2_O_2_ was significantly lower than that of the untreated cells (None).  Figure 2 shows the mRNA levels for proteins related to A*β* production (APP, BACE1, PS1, and PS2) and to degradation (ADAM10, NEP, and ECE1) in the H_2_O_2_-stimulated HLE cells. APP, BACE1, and PS1 mRNAs were increased with increasing concentrations of H_2_O_2_, while PS2 mRNA levels were not altered. The H_2_O_2_ stimulation did not change the mRNA levels of proteins related to A*β* degradation (ADAM10, NEP, and ECE1).

### 3.2. The Preventive Effects of Ferulic Acid on Enhanced A*β* Production and Accumulation in H_2_O_2_-Stimulated HLE Cells

**Figure 3:**
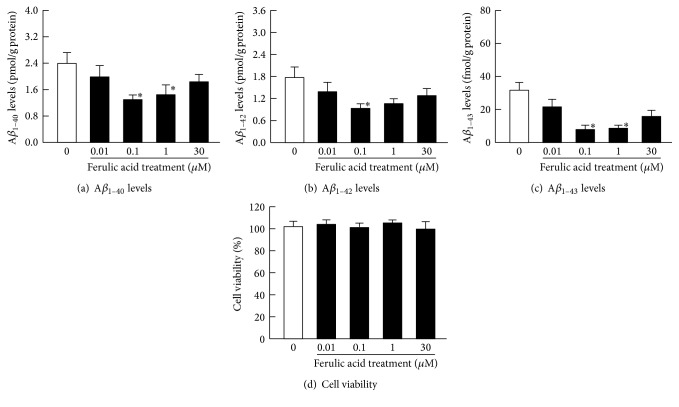
The effect of ferulic acid on A*β*_1–40_ (a), A*β*_1–42_ (b), A*β*_1–43_ (c), and cell viability (d) in 10 *μ*M H_2_O_2_-stimulated HLE cells. The 10 *μ*M H_2_O_2_ and 0–30 *μ*M ferulic acid were added simultaneously to the medium, and the HLE cells were incubated for 24 h. Open columns = H_2_O_2_-stimulated HLE cells. Closed columns = H_2_O_2_-stimulated HLE cells with ferulic acid treatment. The data are presented as the means ± SE of 5–8 experiments. ^*∗*^*P* < 0.05, versus H_2_O_2_-stimulated HLE cells in the absence of ferulic acid (open columns) for each category.

**Figure 4:**
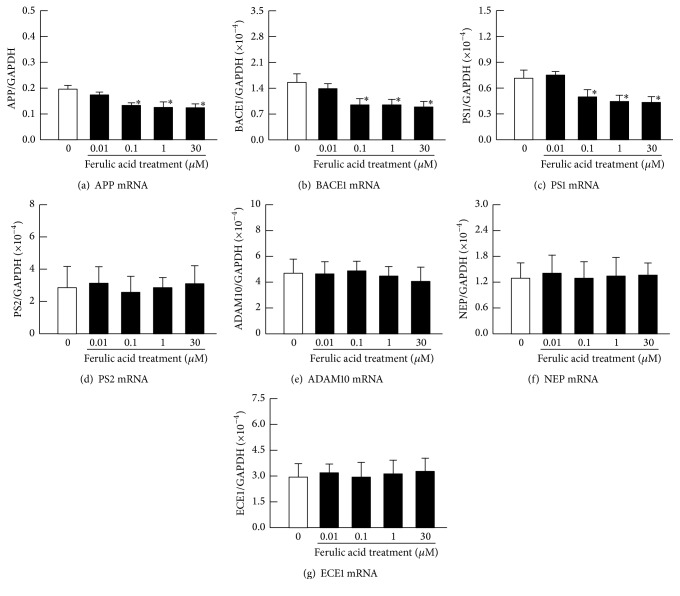
The effect of ferulic acid on mRNA levels of APP (a), BACE1 (b), PS1 (c), PS2 (d), ADAM10 (e), NEP (f), and ECE1 (g) in H_2_O_2_-stimulated HLE cells. The 10 *μ*M H_2_O_2_ and 0–30 *μ*M ferulic acid were added simultaneously to the medium, and the HLE cells were incubated for 24 h. Open columns = H_2_O_2_-stimulated HLE cells. Closed columns = H_2_O_2_-stimulated HLE cells with ferulic acid treatment. The data are presented as the means ± SE of 5–8 experiments. ^*∗*^*P* < 0.05, versus H_2_O_2_-stimulated HLE cells in the absence of ferulic acid (open columns) for each category.

Figures 3(a)–3(c) show the effects of ferulic acid on the specific A*β* levels in the 10 *μ*M H_2_O_2_-stimulated HLE cells. At 0.01–0.1 *μ*M of ferulic acid, A*β* levels were relatively decreased in the H_2_O_2_-stimulated HLE cells. However, at 30 *μ*M of ferulic acid, the A*β* levels were higher than those at 0.1 *μ*M of ferulic acid; thus, the preventive effects of the A*β* level increase peaked at 0.1 *μ*M of ferulic acid.  Figure 3(d) shows the effect of ferulic acid on cell viability in the H_2_O_2_-stimulated HLE cells. A decrease in cell viability was not observed at 0.01–30 *μ*M of ferulic acid.  Figure 4 shows the effects of ferulic acid on the mRNA levels for proteins related to A*β* production (APP, BACE1, PS1, and PS2) and degradation (ADAM10, NEP, and ECE1) in the H_2_O_2_-stimulated HLE cells. Ferulic acid decreased the mRNA levels of APP, BACE1, and PS1. However, the mRNA levels of PS2 and the mRNA levels for proteins related to A*β* degradation (ADAM10, NEP, and ECE1) in HLE cells treated with ferulic acid were similar to the HLE cells without ferulic acid treatment.

### 3.3. The Effects of Ferulic Acid on A*β* Production and Accumulation in HLE Cells

**Figure 5:**
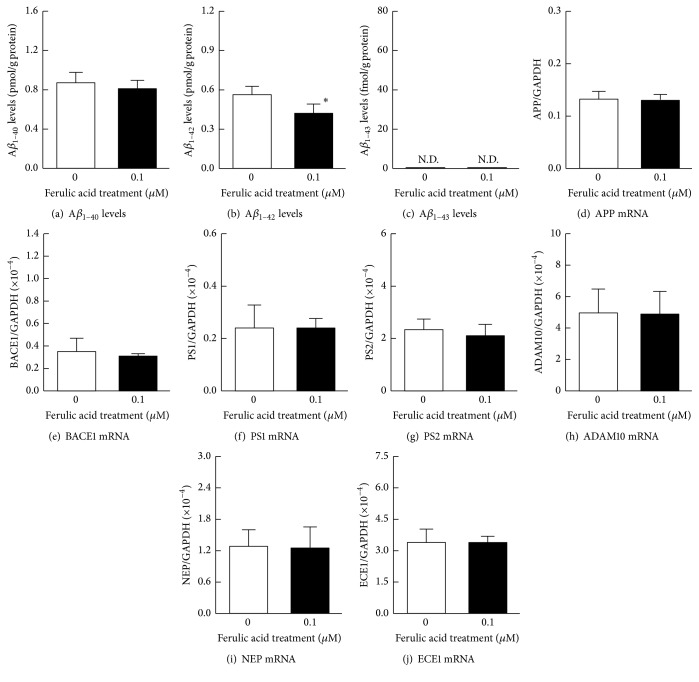
The effect of 0.1 *μ*M of ferulic acid on the production of A*β*_1–40_ (a), A*β*_1–42_ (b), A*β*_1–43_ (c) levels, and mRNA levels of APP (d), BACE1 (e), PS1 (f), PS2 (g), ADAM10 (h), NEP (i), and ECE1 (j) in HLE cells. The 0.1 *μ*M of ferulic acid was added simultaneously to the medium, and the HLE cells were incubated for 24 h. Open columns = the naïve HLE cells. Closed columns = HLE cells in the presence of 0.1 *μ*M ferulic acid. N.D. = not detectable. The data are presented as the means ± SE of five experiments. ^*∗*^*P* < 0.05, versus naïve HLE cells (open columns) for each category.

Because 0.1 *μ*M of ferulic acid showed the greatest suppression of the production of A*β*, we further determined the impact of 0.1 *μ*M of ferulic acid on the HLE cells without H_2_O_2_ stimulation (Figure 5). As shown in Figure 5, the A*β*_1–43_ level was not detected by the HLE cells treated with or without 0.1 *μ*M ferulic acid, and A*β*_1–40_ levels and the mRNA levels of proteins related to A*β* production (APP, BACE1, PS1, and PS2) and degradation (ADAM10, NEP, and ECE1) were similar to those of the naïve HLE cells. On the other hand, the A*β*_1–42_ levels in HLE cells, treated with 0.1 *μ*M ferulic acid, were significantly lower than that without ferulic acid.

## 4. Discussion

In the cataractous lens, an accumulation of A*β* peptide was observed, and lens opacification occurred via oxidative stress [14, 15]. Therefore, the prevention of A*β* accumulation in the lens is important for cataract therapy. In this study, we investigated whether treatments with ferulic acid prevented the A*β* production and accumulation in the human lens and showed that ferulic acid attenuated the increase in the mRNA levels of proteins related to A*β* production (APP, BACE1, and PS1) and prevented the accumulation of A*β*_1–40_, A*β*_1–42_, and A*β*_1–43_ in H_2_O_2_-stimulated HLE cells.

Previous reports showed that the A*β* levels in the retina and brain were enhanced by oxidative stress [19–24]. In addition, we also reported that H_2_O_2_ in the rat lens induced lipid peroxidation and led to the accumulation of A*β*_1–42_ in the lens epithelium [14]. Therefore, in our current study, the HLE cells were exposed to H_2_O_2_ to enhance the A*β* production. The results clearly suggested that 10 *μ*M of H_2_O_2_ induced A*β* production, whereas 0.01–0.1 *μ*M of ferulic acid significantly decreased the mRNAs (APP, BACE1, and PS1) related to A*β* production (Figure 4) and the A*β* production in H_2_O_2_-stimulated HLE cells (Figure 3). It was previously reported that oxidative stress increased the accumulation of A*β* in the lens, retina, and brain [14, 19–24]. In addition, ferulic acid has been shown to possess some scavenging activity for hydroxyl radicals and peroxynitrite [27, 28] and to inhibit the oxidation of substrates. Taken together, our results suggested that 0.1 *μ*M and 1 *μ*M of ferulic acid may prevent H_2_O_2_ effects, resulting in a decrease of specific mRNAs and in A*β* accumulation. However, the mRNA levels of proteins related to A*β* production (APP, BACE1, and PS) in HLE cells incubated with 30 *μ*M of ferulic acid were similar to that of the H_2_O_2_-stimulated HLE cells incubated with 0.1 *μ*M of ferulic acid (Figure 4). However, the A*β*_1–40_, A*β*_1–42_, and A*β*_1–43_ levels in the HLE cells stimulated with 30 *μ*M of ferulic acid were elevated when compared with those of the H_2_O_2_-stimulated HLE cells incubated with 0.1 *μ*M of ferulic acid (Figure 3). It was reported that ferulic acid undergoes specific interactions with A*β* mature fibrils, possibly promoting their destabilization [35]. Moreover, ferulic acid inhibited A*β* aggregation and destabilized preformed A*β* fibrils [36]. From these findings, the destabilization of A*β* fibrils by ferulic acid may be related to the difference in the levels of A*β* in the HLE cells treated with 0.1 *μ*M and 30 *μ*M of ferulic acid. In this study, the A*β* monomer in/on the cells would be measured, since the HLE cells were collected, and all protein fractions were precipitated with 70% formic acid. We tried to measure the A*β* levels in medium; however the A*β* levels were not detected by using ELISA method. We hypothesize that both the fibrils and monomeric A*β* are present in the medium but were below the detection level of the ELISA assay. For the certification of this hypothesis, there is a need to measure the levels of A*β* in the medium by using high resolution mass spectrometry in future studies.

On the other hand, 0.1 *μ*M of ferulic acid shows different effects between A*β*_1–40_ and A*β*_1–42_ levels (Figure 5). It was known that the A*β*_1–42_ was easy to aggregate in comparison with A*β*_1–40_; however, the amount of A*β*_1–42_ production was lower than that of A*β*_1–40_ [7]. Therefore, the destabilization of A*β* aggregation by ferulic acid may strongly affect the A*β*_1–42_ in comparison with A*β*_1–40_. Further studies are needed to elucidate the precise mechanisms for the prevention of A*β* production and the accumulation by ferulic acid in the lens. In addition, it is important to measure the ratio of A*β* fibrils and A*β* monomers to clarify the molecular mechanism involved in the downregulation of APP, BACE1, and PS1 mRNAs in ferulic acid treated cells. Therefore, we are now investigating the protein expression and aggregation of A*β* in H_2_O_2_-stimulated HLE cells incubated with 0.1 *μ*M and 30 *μ*M of ferulic acid by using western blotting and immunostaining methods. Moreover, we are developing a drug delivery system for ferulic acid to the lens. In the future, we will determine the in vivo effects of a ferulic acid ophthalmic formulation on lens opacification and A*β* accumulation in the UPL rat model.

## 5. Conclusions

We have shown that stimulation with H_2_O_2_ leads to increased mRNA levels of proteins related to A*β* production (APP, BACE1, and PS) and to an enhanced accumulation of A*β*_1–40_, A*β*_1–42_, and A*β*_1–43_ in HLE cells. In addition, we showed that treatments with ferulic acid attenuated the increases in the mRNA levels of proteins related to A*β* production and prevented A*β*_1–40_, A*β*_1–42_, and A*β*_1–43_ accumulation in H_2_O_2_-stimulated HLE cells. An ophthalmic eye drop formulation containing ferulic acid could prevent both oxidative stress and A*β* accumulation in HLE cells, resulting in the suppression of lens opacification during cataract. This study provides significant information that can therefore be used to design further studies aimed at developing anticataract drugs.
